# Bioactive Compounds in Cornelian Cherry Vinegars

**DOI:** 10.3390/molecules23020379

**Published:** 2018-02-10

**Authors:** Joanna Kawa-Rygielska, Kinga Adamenko, Alicja Z. Kucharska, Narcyz Piórecki

**Affiliations:** 1 Department of Fermentation and Cereals Technology, Faculty of Food Science, 51-630 Wrocław, Poland; kinga.adamenko@upwr.edu.pl; 2 Department of Fruit, Vegetable and Plant Nutraceutical Technology, Faculty of Food Science, Wroclaw University of Environmental and Life Sciences, 51-630 Wroław, Poland; alicja.kucharska@upwr.edu.pl; 3 Arboretum and Institute of Physiography in Bolestraszyce, 37-700 Przemyśl, Poland; narcyz360@gmail.com; 4 Faculty of Physical Educaion, University of Rzeszów, 35-959 Rzeszów, Poland

**Keywords:** Cornelian cherry vinegar, fermentation, iridoids, polyphenols, antioxidative activity

## Abstract

We analyzed the effect of Cornelian cherry varieties differing in fruit color (‘Yantaryi’—yellow fruits, ‘Koralovyi’—coral fruits, ‘Podolski’—red fruits) and the production method on the physicochemical and antioxidative properties of Cornelian cherry vinegars, and on their content of iridoids and polyphenols. Acetic fermentation was conducted by two methods: I) single-stage (spontaneous) acetic fermentation, without inoculation with microorganisms, and II) two-stage fermentation in which the first stage involved the use of *Saccharomyces bayanus*—Safspirit fruit yeast for alcoholic fermentation, and the second one included spontaneous acetic fermentation. Acetic acid, glycerol, individual iridoids, phenolic acids, flavonols, and anthocyanins were quantified by a high-performance liquid chromatography (HPLC) method. The antioxidative activity was determined based on the following tests: 2,2-Diphenyl-2-picryl-hydrazyl (DPPH^•^), 2,2′-Azino-bis(3-ethylbenzo-thiazoline-6-sulfonic acid (ABTS^•+^), and ferric reducing antioxidant power (FRAP), while the total polyphenols content was determined using the Folin-Ciocialteu (F-C) reagent test. Both the Cornelian cherry variety and vinegar production method affected the antioxidative properties as well as concentrations of iridoids and polyphenols in the finished product. The concentration of total polyphenols (F-C) in vinegars ranged from 326.60 to 757.27 mg gallic acids equivalents (GAE)/100 mL vinegar, whereas the antioxidative activity assayed with the DPPH^•^ and FRAP methods was the highest in the vinegars produced from the coral and red varieties of Cornelian cherry with the two-stage method. Loganic acid predominated among the identified iridoids, reaching a concentration of 185.07 mg loganic acid (LA)/100 mL in the vinegar produced in the two-stage fermentation from the coral-fruit variety. Caffeoylquinic acid derivatives were the main representatives among the identified phenolic compounds. The results of this study demonstrate Cornelian cherry vinegars to be rich sources of biologically-active iridoids and phenolic compounds with antioxidative properties.

## 1. Introduction

Vinegar is a popular food product manufactured in a two-stage fermentation process from a variety of raw materials, mostly including fruits. Many studies conducted so far have addressed fruit vinegar production via fermentation of juices from: apples, strawberries or pomegranates, and also from other untypical raw materials, like: Korean black raspberry or *Litchi Chinensis* fruit tree [1,2,3,4]. Ample investigations have demonstrated fruit vinegars to possess strong antioxidative properties and, thereby, to serve protective functions against adverse effects of pathogenic enteral flora, to exhibit anti-diabetic potential, to reduce blood concentration of lipids, to prevent arterial hypertension or to decrease the glycemic index of food products being sources of carbohydrates. Vinegars may as well be applied in the production of medicinal preparations being less straining to the body [5,6,7,8,9]. The strong antioxidative effect of vinegars is due to their bioactive compounds including: carotenoids, phytosterols and also phenolic compounds [10,11] represented by, among others, flavonoids, tannins, anthocyanins or phenolic acids [12]. 

Fruit of Cornelian cherry (*Cornus mas* L.) contain a wide spectrum of compounds with antioxidative properties. They are used to produce juices, syrups and jams, but also—increasingly often—to prepare a fruit liquor (the so-called “dereniówka”) which is an alcoholic distillate from these fruits. Previous studies have demonstrated both extracts and substances prepared from Cornelian cherry fruits to be rich in phenolics compounds and iridoids. The latter are representatives of cyclopentane monoterpenes with the main backbone constituted by a cyclopentane ring and pyran, which occur mainly in the glycosidic form. They display hypotensive, antibiotic, and anti-inflammatory effects [13,14].

The quality of vinegars is determined by a few basic factors, the key ones of which include the raw materials they are made of, their method of acetylation, and their ageing procedure. Positive health outcomes of fruit vinegar consumption may be associated with transformations proceeding during their fermentation which lead to an increase in the content of their biologically-active compounds and to a change in their polyphenolic profile [14,15]. Earlier investigations have shown that contents of polyphenols in fruits and fruit products depend on both their pre-treatment and production methods and on conditions of their storage [14,16,17]. No works have been found in the available literature on the characteristics and properties of vinegars made from Cornelian cherry fruits. In this manuscript, we present results of analyses of the effect of the applied Cornelian cherry variety and fermentation method on the concentrations of iridoid and phenolic compounds and on the antioxidative properties of Cornelian cherry vinegars.

## 2. Results

### 2.1. Dynamics of Alcoholic Fermentation

**Figure 1:**
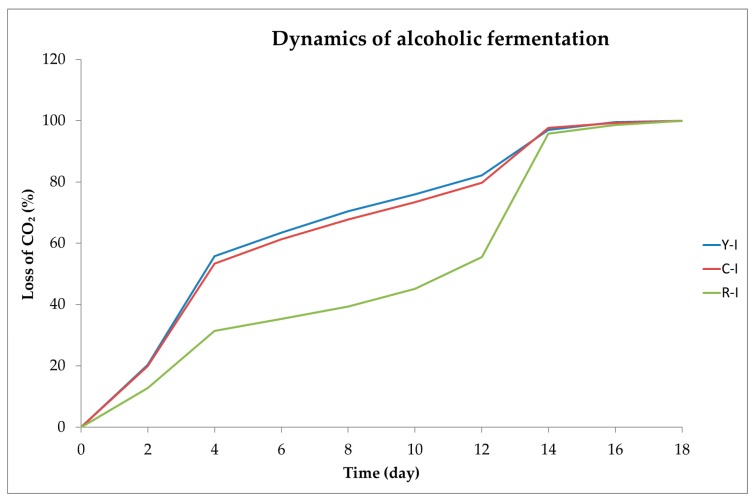
Dynamics of single-stage alcoholic fermentation (I) of juices made of three varieties of Cornelian cherry differing in fruit color: yellow (Y), coral (C), and red (R), expressed as the percentage loss of carbon dioxide (CO_2_) evolved within 18 days of the fermentation process.

Dynamics of the alcoholic fermentation process of juices from Cornelian cherry fruits of different coloration (yellow fruits − Y, coral fruits − C and red fruits −R) was determined based on changes in the weight of fermentation samples in time and expressed as the percentage loss of CO_2_. Results of these determinations for the single-stage fermentation are presented in Figure 1. 

Dynamics of the single-step fermentation process of juices made of yellow (YI) and coral (CI) Cornelian cherries was similar and significantly higher than the fermentation dynamics of juice pressed from the red-fruit variety (RI). This observation was confirmed by the statistical analysis results. During the fermentation process, the volume of CO_2_ emitted from juice made of red fruit (RI) Cornelian cherry was by ca. 30% lower than in the other analyzed juices.

### 2.2. Extract Content, pH Value, and Concentrations of Acetic Acid, Alcohol and Glycerol

Extract content and concentrations of acetic acid, glycerol and ethyl alcohol in the prepared vinegars and their pH values are presented in Table 1. Extract content ranged from 8.0 brix degrees (°Bx) in YII sample to 12.8 °Bx in CI sample, however these differences were statistically insignificant. The lowest pH value was measured in vinegars produced by the two-stage process from yellow (YII) and red (RII) fruits, whereas the highest one in the vinegar made of coral Cornelian cherry fruits (CI) in the single-stage fermentation process. Both the extract content and pH value were lower in the vinegars produced in the two-stage than in the single-stage process. Concentrations of acetic acid, glycerol, and ethyl alcohol in the analyzed vinegars were determined using high-performance liquid chromatography (HPLC). The concentration of acetic acid exceeded 40 g/L vinegar in all samples. The highest acidity, indicated by 47.06 g acetic acid/L vinegar, was found for the vinegar produced from yellow-fruit Cornelian cherry in the YII, whereas the lowest one for the vinegar made of fruits of the same variety but in the single-stage fermentation (YI). In the case of yellow-fruit and red-fruit varieties, a higher concentration of acetic acid was determined in the vinegars produced in the two-stage than in the single-stage process of fermentation. The highest concentration of ethyl alcohol, i.e., 7.46 g/L vinegar, was assayed in the vinegar produced from coral-fruit Cornelian cherry in the single-stage fermentation (CI). In contrast, no ethanol was found in the vinegars made of coral and red fruits in the two-stage process (CII and RII). The lowest concentration of ethanol, reaching 1.79 g/L vinegar, was determined in the vinegar produced from coral-fruit Cornelian cherry in the single-stage fermentation (CI). Glycerol was found in all vinegar samples and its concentration ranged from 0.98 to 2.12 g/L vinegar depending on the applied variety of Cornelian cherry and vinegar production method. Its highest concentration was determined in the vinegar made of red-fruit Cornelian cherry in the two-stage fermentation process.

### 2.3. Color

Values of color parameters L*, a* and b* determined in vinegars produced from juices made of different varieties of Cornelian cherry are summarized in Table 2. Vinegars produced via single-stage acetic fermentation had a significantly higher value of color parameter L*, indicative of sample lightness. The two-stage production process caused significant darkening of the vinegars. In turn, values of color parameters a* and b* were higher in the case of the vinegars produced in the two-stage process, compared to these manufactured via the single-stage fermentation.

An exception was the value of a* parameter determined in the vinegar from red-fruit Cornelian cherry (RI). For example, values of color parameters a* and b* of the vinegar made of yellow-fruit variety in the single-stage process (YI) reached 10.98 and 48.22, respectively, whereas in the vinegar produced from the same variety of Cornelian cherry but in the two-stage process (YII) they accounted for 15.07 and 52.64, respectively.

### 2.4. Concentration of Total Polyphenols and Antioxidative Activity

**Figure 2:**
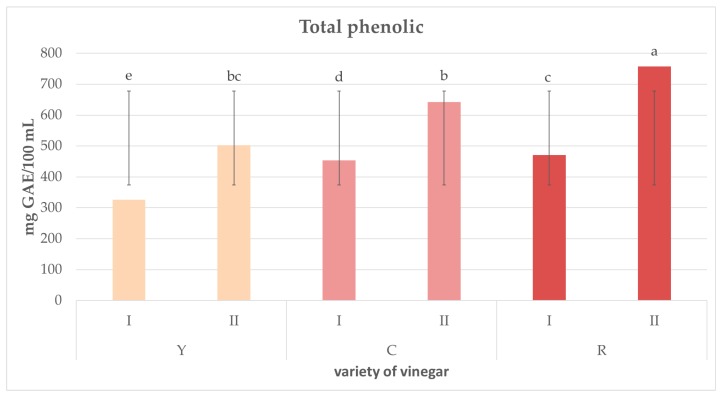
Total polyphenols concentration in vinegars produced from juices made of three varieties of Cornelian cherry differing in fruit color (Y, C, R) by using two different methods of fermentation (I, II). Values are expressed as the mean (*n* = 3) ± standard deviation. Mean values with different letters (a, b, c, etc.) are statistically different (*p*-value < 0.05).

Figure 2 presents the results of analyses of total polyphenols in vinegars from three varieties of Cornelian cherry differing in fruit color. The highest concentration of polyphenols (757.27 mg gallic acids equivalents (GAE)/100 mL vinegar) was determined in the vinegar produced from red-fruit Cornelian cherry in the two-stage fermentation process (RII). In contrast, their lowest concentration accounting for 326.60 mg GAE/100 mL vinegar was found in the vinegar made of yellow-fruit variety in the single-stage process (YI). Results obtained demonstrate the total content of polyphenols in the analyzed vinegars to depend on vinegar production method and color of fruits, i.e., their variety. Regardless of Cornelian cherry variety the juice was made of, the total concentration of polyphenols was significantly higher in the vinegars produced in the two-stage fermentation process, compared to these manufactured in the single-stage process.

**Figure 3:**
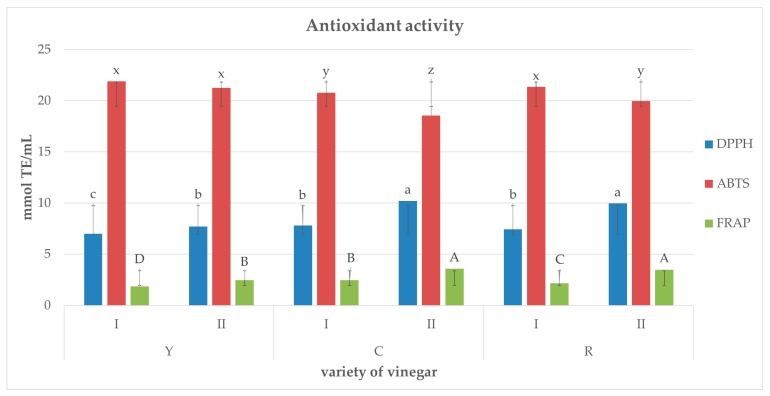
Antioxidative activity (FRAP, DPPH^•^, ABTS^•+^) of vinegars produced from juices made of three varieties of Cornelian cherry differing in fruit color (Y, C, R) by using two different methods of fermentation (I, II).^1^ Values are expressed as the mean (*n* = 3) ± standard deviation. Mean values with different letters: A, B, C, D (FRAP); a, b, c (DPPH^•^); x, y, z (ABTS^•+^) are statistically different (*p* < 0.05).

Figure 3 depicts the antioxidative activity of the analyzed vinegars determined with three methods: ferric reducing antioxidant power (FRAP), 2,2-Diphenyl-2-picryl-hydrazyl (DPPH^•^), and 2,2′-Azino-bis(3-ethylbenzo-thiazoline-6-sulfonic acid (ABTS^•+^). In the case of DPPH^•^ and FRAP assays, the strongest antioxidative properties were shown for the samples produced via two-stage fermentation of juice from red-fruit (RII) and coral-fruit (CII) Cornelian cherry, i.e.,: 10.23 mmol trolox (TE)/mL and 9.97 mmol TE/mL respectively when assayed with the DPPH^•^ method, as well as 3.6 mmol TE/mL and 3.5 mmol TE/mL respectively when assayed with the FRAP method. The lowest antioxidative activity determined with these methods was found for the vinegar produced from yellow-fruit variety in the single-stage acetic fermentation was demonstrated in vinegars produced in the two-stage compared to the single-stage fermentation. No similar tendency was observed in the case of the ABTS^•+^ method. The activity against ABTS^•+^ cation radical in the vinegars from coral-fruit and red-fruit varieties was higher when they were produced in the two-stage fermentation process, whereas in the case of vinegars made of yellow-fruit variety there were no significant differences between fermentation processes. There were linear correlations between total phenolic content and antioxidant activity.

### 2.5. Quantitative Identification of Iridoids and Phenolic Compounds

The vinegars produced from Cornelian cherry were found to contain compounds representative of monoterpenes (iridoids) and polyphenols (flavonols, anthocyanins, phenolic acids) (Table 3). The identified iridoids included: loganic acid (LA), cornuside (Co.) and a combination of sweroside (S) and loganin (Lo). Loganic acid was the predominating iridoid and its content in the analyzed vinegars ranged from 73 to 86% of total iridoids. In turn, Co. content constituted from 6 to 15% of total iridoids. The highest concentrations of LA and Co. were determined in the vinegar produced from the coral-fruit variety of Cornelian cherry in the two-stage fermentation (CII), i.e.,: 190.75 and 35.04 mg/100 mL vinegar, respectively. The highest concentration of S + Lo, accounting for 34.72 mg/100 mL vinegar, was determined in the vinegar made of red-fruit variety in the two-stage process (RII). In the case of all identified iridoids, their higher concentrations were found in the vinegars produced in the two-stage process in which *Saccharomyces bayanus*—Safspirit fruit yeast were used for alcoholic fermentation, compared to the vinegars obtained via the single-stage fermentation. Compounds identified in the group of phenolic acids included derivatives of caffeoylquinic acid (CQA d) and *p*-coumaric acid (*p*-CA d) and ellagic acid (EA). The predominating compound in this group was CQA d (9.64–24.18 mg/100 mL), followed by *p*-CA d (3.50–10.85 mg/100 mL), and EA (0.62–1.99 mg/100 mL). 

The highest concentrations of *p*-CA d and CQA d reaching 24.18 and 10.85 mg/100 mL vinegar respectively, were determined in the vinegar produced from the coral-fruit variety in the two-stage fermentation process. In turn, the highest concentration of EA, i.e., 1.99 mg/100 mL vinegar, was found in the vinegar produced from the red-fruit variety in the two-stage process (RII). Among the identified flavonols, aromadendrin 7-*O*-glucoside (A 7-glu) was determined only in the vinegars from red-fruit variety of Cornelian cherry and its higher concentration accounting for 3.35 mg/100 mL vinegar was found in the sample produced in the two-stage process (RII). Kaempferol 3-*O*-galactoside (Kp 3-gal) was detected in the vinegars from the red-fruit and coral-fruit varieties of Cornelian cherry. Its concentration ranged from 0.10 to 2.53 mg/100 mL vinegar and was higher in the vinegars from the red-fruit variety. Other identified flavonols were: quercetin 3-*O*-galactoside (Q 3-gal) and quercetin 3-*O*-glucuronide (Q 3-gluc). The highest Q 3-gal concentration was found in the vinegars from the red-fruit and coral-fruit varieties produced in the two-stage fermentation (RII, CII), and reached: 0.59 mg/100 mL of RII vinegar and 0.58 mg/100 mL of CII vinegar. In turn, the highest concentration of Q 3-gluc was assayed in the sample of vinegar produced from coral-fruit Cornelian cherry in the two-stage process (CII). Its concentration in the analyzed vinegars ranged from 2.63 mg/100 mL in RI to 6.00 mg/100 mL in CII. Four anthocyanins were identified in the analyzed vinegars, but only in these produced from the red-fruit variety. Concentrations of all identified anthocyanins were higher in the samples obtained with the two-stage method (RII) and accounted for (in 100 mL vinegar): 0.75 mg cyanidin 3-*O*-galactoside (Cy 3-gal), 0.41 mg cyanidin 3-*O*-robinobioside (Cy 3-rob), 1.69 mg pelargonidin 3-*O*-galactoside (Pg 3-gal), and 0.30 mg pelargonidin 3-O-robinobioside (Pg 3-rob).

## 3. Discussion

Many researchers have recently paid special attention to fruits of Cornelian cherry (*C. mas* L.) [18,19,20], that are rich in biologically-active compounds, iridoids in particular, and thereby exhibit potential therapeutic properties [21,22,23]. 

Brix degrees are indicative of the percentage content of soluble substances, including mainly sugars, proteins and mineral salts. However, in such products as vinegars, they are used to determine the content of sugars only (with no consideration given to other soluble compounds). Factors which may determine the °Bx value of vinegars include: the culture of microorganisms used for the fermentation process and raw material used for vinegar production. The °Bx value is strictly linked with the fermentation process as sugar content decreases along with an increasing fermentation activity of microorganisms and may thus greatly vary [24]. The °Bx values determined in our study for Cornelian cherry vinegars were similar to extract content in fruit vinegars analyzed by other scientists [24,25]. The pH value of fruit vinegars is directly dependent on the raw materials they were made of [25]. Earlier investigations addressing the characteristics of the physicochemical properties of fruit vinegars have demonstrated their pH values to fit within the similar ranges [25,26,27].

Although the standard method for vinegar production is highly time-consuming, it enables the maximal consumption of substrates to produce appropriate amount of ethanol in the first stage of production and of acetic acid in the finished product [28]. The acidity of vinegars made of various raw materials should be not lesser than 4% [29]. In turn, ethyl alcohol is a substrate of acetic fermentation and its concentration in vinegars is low [30]. Results obtained in our study are consistent with those reported by other authors about ethyl alcohol and acetic acid contents in fruit vinegars [25,26,27,28,31,32,33]. The alcoholic fermentation of sugars results in the generation of by-products, including glycerol. Earlier investigations have shown from 0.23 to 0.56% of glycerol in apple vinegars [28]. Compared to the above results, the concentration of glycerol in the analyzed Cornelian cherry vinegars was lower and ranged from 0.99 to 2.12 mg/100 mL.

The color of vinegars is an important factor which affects purchase decisions made by consumers [34]. The study addressing color analysis of vinegars produced from fruits of, among others, hawthorn, grape, sour cherry, pomegranate and strawberries has shown that values of color parameters depend on fruit species [25].

The Cornelian cherry vinegars analyzed in our study differed in the concentration of total polyphenolics which ranged from 326.60 to 757.27 mg GAE/100 mL. No works have been found in the available literature that would report such a high concentration of total polyphenolics in fruit vinegars. Earlier studies have shown total polyphenolics content to reach 255 mg GAE/100 mL in balsamic vinegar [26], 222.88 mg GAE/100 mL in apple vinegar [25], 161 mg GAE/100 mL in strawberry vinegar [9], and 138 mg GAE/100 mL in citrus fruit vinegar [35]. Significantly lower concentrations of these compounds were determined in vinegars made of grape—26 mg GAE/100 mL [36] or hawthorn—28 mg GAE/100 mL [26]. Considerable differences observed between results obtained in our study and findings of other authors may be due to the fact that the concentration of polyphenolics in vinegars depends most of all on the type of raw material as well as on their production process including: duration and conditions of fermentation and conditions of ageing [36,37]. The content of total polyphenolics in fruits of Cornelian cherry expressed as gallic acid equivalents ranges from 209 to 811 mg GAE/100 g [38,39,40]. In other fruits, it is lower compared to Cornelian cherry and accounts for: 72.27–290 mg GAE/100 g in grapes [41,42,43], 398–670.9 mg GAE/100 g in berries [43,44,45], 109.72–268.35 mg GAE/100 g in goji berries [46,47], or 260–2556 mg GAE/100 g in chokeberry fruits [34,48,49,50]. Samples subjected to ethanolic fermentation with the use of *S. bayanus*—Safspirit fruit yeast were characterized by higher concentrations of polyphenolics, which may result from the fact that alcoholic fermentation stabilizes the antioxidative potential of plant materials relatively well, which is due to the low redox potential during anaerobic fermentation of worts.

The total content of polyphenolics is associated with antioxidative properties. Hence, the antioxidative activity determined with FRAP and DPPH^•^ assays was higher in the vinegars produced in the two-stage fermentation process. Alongside results of total polyphenolics concentration, the strongest antioxidative properties were determined in the vinegars made of red-fruit and coral-fruit varieties of Cornelian cherry. The analyzed Cornelian cherry vinegars had a higher antioxidative activity compared to fruit vinegars obtained by other researchers. It may be due to the fact that Cornelian cherry fruits are characterized by a higher content of polyphenolics compared to other fruit materials used for vinegar production, as the total content of polyphenolics affects the antioxidative activity. Earlier investigations addressing antioxidative properties of fruit vinegars were conducted using the FRAP method [26,51,52], the DPPH^•^ method [26,53,54,55] and also the ABTS^•+^ method [26,27,51,52,53,54,55], which shown that the antioxidant activity of vinegars depends on the type of fruit.

The analyzed Cornelian cherry vinegars were characterized by various concentrations of bioactive compounds. The predominating group of these compounds were iridoids whose total concentration ranged from 173.3 to 253.2 mg/100 mL. Iridoids occur only in a few fruits, including Cornelian cherry and Japanese cornel, blue honeysuckle, lingonberry or cranberry [13,14,56,57], hence compounds belonging to this group have not been detected in fruit vinegars studied so far. The presence of iridoids in consumed food products is important as they affect their taste and biological properties. Iridoids with an open cyclopentane ring between C-7 and C-8 atom of carbon, referred to as secoiridoids, are responsible for the bitter taste, like e.g., oleuropein in olives. No distinct bitterness may be perceived in Cornelian cherry fruits in spite of the fact that their iridoids include open-ring compounds like e.g., cornuside and secologanin [58]. This is also the case with Cornelian cherry products like jams and liqueurs [13] or vinegars, like these analyzed in our study. The presence of iridoids in food products increases their biological value, because they exhibit, i.e., anti-inflammatory, antimicrobial, antidiabetic, anti-atherosclerotic, cyto-, hepato-, neuro- and renal-protective, antiplatelet, and antiglaucomic activities [21,22,23,59]. The quality and biological properties of plant food products are also affected by polyphenolics, which have especially great impact on their color, astringency, flavor, and antioxidative activity [60]. The predominating phenolic compounds in the analyzed vinegars were phenolic acids. Compared to Cornelian cherry fruits [13], the fruit vinegar was characterized by richer quantitative and qualitative composition of phenolic acids, which suggests that these acids may be released from high-molecular structures during fermentation. High concentrations of such phenolic acids as: gallic acid, *p*-coumaric acid, caffeic acid, and caffeoylquinic acid, were determined in fruit vinegars also by other authors [26,51,55]. The major flavonoids of Cornelian cherry fruits include anthocyanins, followed by flavonols. Anthocyanins are highly unstable pigments and occur only in strongly colored varieties of Cornelian cherry, hence their trace amounts were identified only in the vinegar from the red-fruit variety. In turn, flavonols were determined in all analyzed vinegars, regardless of variety, and their qualitative composition was comparable with the composition of fruits. The concentration of flavonols in vinegars is affected by their content in fruits. Kelebek et al. determined concentrations of flavonols in apple and grape vinegars at 2.46 mg/100 mL and 0.07 mg/100 mL, respectively [55]. The analysis of our results demonstrates that the content of flavonols is influenced not only by the species but also by the variety of fruits, because the highest concentration of these compounds was determined in vinegars from red fruits and the lowest one in vinegars from yellow fruits of Cornelian cherry.

## 4. Materials and Methods

### 4.1. Reagents and Standards

1,1-Diphenyl-2-picrylhydrazyl radical (DPPH^•^); 6-hydroxy-2,5,7,8-tetramethylchroman-2-carboxylic acid (Trolox); 2,4,6-tri(2-pyridyl)-s-triazine (TPTZ), dimethyl sulfoxide (DMSO), FeCl_3_, acetonitrile, formic acid, and S, sulfuric acid and sodium hydroxide were acquired from Sigma-Aldrich (Steinheim, Germany). Acetic acid was obtained from Chempur (PiekarySląskie, Poland). Acetonitrile for liquid chromatography–mass spectrometry was purchased from POCh (Gliwice, Poland). Loganic acid, and Lo, *p*-CA, 5-*O*-caffeoylquinic acid (5-CQA, chlorogenic acid), quercetin 3-*O*-glucoside, kaempferol-3-*O*-glucoside (Kp 3-gluc), cyanidin 3-*O*-glucoside (Cy 3-glc) were purchased from Extrasynthese (Lyon Nord, France). All reagents were of analytical grade.

### 4.2. Biological Material

The biological material used for the ethanolic fermentation of Cornelian cherry fruit were *S. bayanus*—Safspirit fruit yeast from the Fermentis company (Lesaffre, Marcq-en-Barœul, France). Before being inoculated, dried yeast obtained from the producer were rehydrated in distilled water at a temperature of 25 °C for 30 min.

### 4.3. Raw Material

Cornelian cherry fruits (*C. mas* L.) from 3 cultivars: ‘Florianka’ (red color), ‘Yantarnyi’ (yellow color) and ‘Koralovyi’ (coral color) were harvested in the Arboretum in Bolestraszyce, near Przemyśl, Poland. The plant materials were authenticated by Prof. Jakub Dolatowski (Arboretum and Institute of Physiography in Bolestraszyce, Przemyśl, Poland), and the adequate voucher specimens (‘Yantarnyi’—BDPA 14131; ‘Koralovyi’—BDPA 14136; ‘Podolski’—BDPA 10462) have been deposited at the Herbariums of Arboretum in Bolestraszyce, Poland. Fruits were harvested in September 2016, and immediately frozen at −20 °C.

### 4.4. Preparation of Fermentation Samples

All fruits of Cornelian cherry were pressed through a laboratory press to obtain juices that were further used in the study. Resultant juices were poured into fermentation flasks. In the first experimental variant involving the spontaneous fermentation process, the flasks were prepared for fermentation by covering them with a sterile gauze to control the access of oxygen. In turn, in the second experimental variant which included two-stages, first of which consisted in alcoholic fermentation, the juices were inoculated with *S. bayanus*—Safspirit fruit yeast in the amount of 0.5 g/L. The flasks were tightly closed with fermentation bungs to produce anaerobic conditions. In all experimental variants, pH was adjusted to 4.5 using 0.1 M NaOH before alcoholic fermentation.

### 4.5. Fermentation

Table 4 presents symbols used to describe experimental variants.

The samples prepared for single-stage fermentation (YI, CI, RI) were placed on a 358 A Type laboratory shaker (Elpin Plus, Lubawa, Poland) and mixed with the frequency of 50 cycles/min at a temperature of 25 °C for 60 days. In turn, the samples intended for the two-stage process of vinegar production (YII, CII, RII) were firstly subjected to alcoholic fermentation with *S. bayanus*—Safspirit fruit yeast under anaerobic conditions, at a temperature of 25 °C for 18 days. Afterwards, the fermented juices were decanted from above the yeast precipitate and prepared for the next stage like the samples intended for the spontaneous fermentation. The second-stage of fermentation lasted 42 days.

### 4.6. Analytical Methods

#### 4.6.1. Extract, pH, Acetic Acid, Ethanol and Glycerol Content

Extract (°Bx) in vinegars was tested at the temperature of 20 °C with the use of a Densito 30PX densimeter (Mettler Toledo, Columbus, OH, USA) whereas pH was measured with the use of a Mettler Toledo MP 240 pH-meter. 

Acetic acid, ethanol and glycerol were determined by means of HPLC [61]. Degassed and centrifuged (2675 centrifugal force (RCF), 6000 rpm, 10 min) samples were diluted with ultra-pure water in the volumetric ratio of 1:7; then filtered through a nylon syringe filter with pore size of 0.22 μm into chromatographic vials. The samples were analyzed using a Prominence liquid chromatography system (Shimadzu Corp., Kyoto, Japan) equipped in a Rezed ROA-Organic Acid H+ column (300 × 4.6 mm) from Phenomenex (Torrance, CA, USA). The following parameters of measurements were applied: injection volume 20 μL, elution temperature 60 °C, flow rate 0.6 mL/min, mobile phase 0.005 M H_2_SO_4_, and thermostat refractometric detector at 50 °C. Concentrations of acetic acid, ethyl alcohol and glycerol were determined based on a five-point calibration curve integrated in Chromax 10.0 software by Pol-Lab (Wilkowice, Poland).

#### 4.6.2. Instrumental Analysis of Color

The color of the vinegars (reflectance values: L*, a* and b*) was measured using a Color Quest XE HunterLab (Reston, VA, USA) spectrophotometer. Each sample was placed in a glass cuvette, and its color L*, a*, b* values were determined using Illuminant D65 and an observer angle of 10°. L* denotes ‘lightness’, and its values range from 0 to 100 (0 for ideal black, and 100 for ideal white). A positive value of a* indicates ‘red color’, a negative value of a* indicates ‘green color’, a positive value of b* indicates ‘yellow color’, and a negative value of b* indicates ‘blue color’.

#### 4.6.3. Phenolic Compound Analysis

Total polyphenol content and antioxidant activity were analyzed with the use of a UV-2401 PC spectrophotometer (Shimadzu Corp).

##### Determination of Total Polyphenols Content

The total polyphenols content of the vinegars was determined using the Folin-Ciocalteu (F-C) sprectophotometric method [62]. Vinegar samples and F-C reagent were pipetted into cuvettes. After 3 min, 1 mL of a 20% aqueous solution of sodium carbonate (Na_2_CO_3_) and 2 mL of distilled water were added. The absorbance was measured at 7650 nm after 1 h, and the results were expressed as mg of GAE per 100 mL of vinegar. Data were expressed as the mean value for three measurements. Calibration curves of gallic acid in the range 0.30–9.00 mg GAE/L were used to read off the results.

##### Free-Radical-Scavenging Ability by the Use of a DPPH Radical

The antiradical activity was determined using a DPPH^•^ assay [63]. 0.1 mL samples of vinegars were mixed with 2 mL of 0.04 mmol/L DPPH^•^ in methanol and 0.4 mL of H_2_O. After 10 min of incubation at room temperature, the absorbance was measured with a spectrophotometer at 517 nm using disposable polystyrene cuvettes. A calibration curve was prepared with Trolox solution (0.05 × 10^−1^ mmol/L). The data were expressed as Trolox equivalent (TE) of antioxidative capacity per hundred milliliters of the vinegar (TEAC, mmol TE/mL). All measurements were performed in triplicate. Calibration curves, in the range 2–10 µmol TE/L, showing good linearity (*r*^2^ ≥ 0.998).

##### Free-Radical-Scavenging Ability by the Use of a ABTS Radical Cation.

The antioxidative activity of vinegars was determined using the ABTS^•+^ assay [64]. 0.03 mL samples of vinegar were mixed with 3 mL of ABTS^•+^ solution with measured absorption of 0.700 at a wavelength of 734 nm. After 6 min the absorbance of samples was measured. Each sample was tested in triplicate. The data were expressed as mmol Trolox equivalent of antioxidative capacity per 100 milliliters of the vinegar (mmol TE/mL). Calibration curves, in the range 1.70–21.70 µmol TE/L, showed good linearity (*r^2^* ≥ 0.999).

##### Ferric Reducing/Antioxidant Power (FRAP) Assay

The FRAP is based on the reduction of ferric 2,4,6-tris(2-pyridyl)-1,3,5-triazine [Fe(III)-TPTZ] to the ferrous complex at low pH, followed by spectrophotometric analysis [65]. Briefly, the reagent was prepared by mixing 10 mmol 2,4,6-Tris(2-pyridyl)-s-triazine (TPTZ)/L reagent with 20 mmol/L ferric chloride in acetate buffer (pH 3.6). Quantitative analyses were performed by the external standard method using ferrous sulfate (2 × 10^−1^ mmol/L) as the reference standard and correlating the absorbance (λ 593 nm) with the concentration. 0.2 mL samples of vinegar were mixed in polystyrene cuvettes with 0.8 mL of distilled water and 3 mL of ferric complex. The results were calculated and expressed as millimoles of Trolox per milliliter of the vinegar. The absorbance was read in disposable polystyrene cuvettes using a spectrophotometer. All measurements were performed in triplicate. Calibration curves, in the range 1.25–12.50 µmol TE/L, showed good linearity (*r*^2^ ≥ 0.998).

##### Quantification of Iridoids and Polyphenols by HPLC-PDA

The high-performance liquid chromatography photodiode array detection method (HPLC-PDA) analysis was previously described by Kucharska et al. [30]. It was performed using a Dionex (Germering, Germany) system equipped with an Ultimate 3000 model diode array detector, LPG-3400A quaternary pump, EWPS-3000SI autosampler, TCC-3000SD thermostated column compartment, and controlled by the Chromeleon v.6.8 software (Thermo Scientific Dionex, Sunnyvale, CA, USA). A Cadenza Imtakt C5-C18 column (75 4.6 mm, 5 m) was used (Imtakt, Kyoto, Japan). The mobile phase was composed of solvent A (4.5% aq. formic acid, *v*/*v*) and solvent B (100% acetonitrile). The elution system was as follows: 0–1 min 5% B in C, 20 min 25% B in A, 21 min 100% B, 26 min 100% B, 27 min 5% B in A. The flow rate of the mobile phase was 1.0 mL/min and the injection volume was 20 µL. The column was operated at 30 °C. Iridoids were detected at 245 nm, phenolic acids and their derivatives at 320 nm, ellagic acid at 254 nm, flavonols at 360 nm, and anthocyanins at 520 nm. Loganic acid and Co. were expressed as LA, Lo and S as Lo, caffeoylquinic acids derivatives as 5-*O*-caffeoylquinic acid, *p*-cumaric acid derivatives as *p*-cumaric acid, quercetin derivatives and aromadendrin 7-*O*-glucoside as quercetin 3-*O*-glucoside, kaempferol 3-*O*-galactoside as kaempferol 3-*O*-glucoside, anthocyanins as cyanidin 3-*O*-glucoside. The results were expressed as mg per 100 mL vinegar.

### 4.7. Statistics

Mean deviations are shown on graphs. Selected data were processed using the Statistica 13.5 software (StatSoft, Tulsa, OK, USA), a one-way analysis of variance (ANOVA) at a significance level of α = 0.05. Differences between means were tested with the Duncan test (*p*-value < 0.05).

## 5. Conclusions

The study demonstrated the feasibility of using the analyzed varieties of Cornelian cherry for the production of fruit vinegars with potential health properties. The highest concentration of biologically-active compounds was determined in the vinegar made of red-fruit variety. Cornelian cherry juices allow one to obtain vinegars rich in iridoids. In addition, this study proved the fermentation method to have a significant impact on the final concentration of active compounds in the finished product. The use of *S. bayanus—*Safspirit fruit yeast to carry out alcoholic fermentation significantly increased the content of biologically-active compounds in the final products. Cornelian cherry vinegars, as a natural food product, can be a good source of antioxidants in the human diet.

## Figures and Tables

**Table 1:** Extract content (brix degrees (°Bx)), pH value, and concentrations of acetic acid (g/L), ethyl alcohol (g/L) and glycerol (g/L) in vinegars produced from juices made of three varieties of Cornelian cherry differing in fruit color (Y, C, R) by using two different methods of fermentation (I, II).

Variety of Cornelian Cherry Fruit	Method of Fermentation	Extract (°Bx)	pH	Glycerol (g/L)	Alcohol (g/L)	Acetic Acid (g/L)
Y ^1^	I ^2^	10.00 ^a^ ± 0.28	2.81 ^b^ ± 0.01	0.99 ^f^ ± 0.01	1.79 ^d^ ± 0.01	41.65 ^f^ ± 0.23
	II	8.00 ^a^ ± 0.45	2.76 ^a^ ± 0.01	1.20 ^e^ ± 0.04	3.77 ^b^ ± 0.01	47.06 ^a^ ± 0.44
C	I	12.80 ^a^ ± 0.28	2.90 ^d^ ± 0.01	1.56 ^d^ ± 0.03	7.46 ^a^ ± 0.01	42.30 ^d^ ± 0.24
	II	11.50 ^a^ ± 0.71	2.86 ^c^ ± 0.01	1.72 ^c^ ± 0.03	0.00	42.13 ^e^ ± 0.19
R	I	11.40 ^a^ ± 0.57	2.85 ^c^ ± 0.00	1.92 ^b^ ± 0.03	2.3 ^c^ ± 0.01	42.64 ^c^ ± 0.76
	II	9.30 ^a^ ± 0.42	2.76 ^a^ ± 0.01	2.12 ^a^ ± 0.03	0.0	45.38 ^b^ ± 0.04

^1^ Y, ‘Jantarnyi’ with yellow fruits; C, ‘Koralovyi’ with coral fruits; R, ‘Podolski’ with red fruits; ^2^ I, single-step process of fermentation; II, two-step process of fermentation. ^3^ Values are expressed as the mean (*n* = 3) ± standard deviation. Mean values with different letters (a, b, c, etc.) within the same column are statistically different (*p*-value < 0.05).

**Table 2:** Values of color L*, a* and b* in vinegars produced from juices made of three varieties of Cornelian cherry differing in fruit color (Y, C, R) by using two different methods of fermentation (I, II).

Variety of Cornelian Cherry Fruit	Method of Fermentation	L*	a*	b*
Y ^1^	I ^2^	70.73 ± 0.00 ^a,3^	10.98 ± 0.01 ^f^	48.22 ± 0.04 ^d^
	II	64.46 ± 0.01 ^b^	15.07 ± 0.01 ^e^	52.64 ± 0.00 ^c^
C	I	49.85 ± 0.00 ^c^	36.05 ± 0.00 ^c^	59.97 ± 0.02 ^b^
	II	41.06 ± 0.01 ^d^	45.79 ± 0.01 ^b^	61.01 ± 0.13 ^a^
R	I	16.92 ± 0.01 ^e^	46.67 ± 0.00 ^a^	9.39 ± 0.10 ^e^
	II	5.72 ± 0.26 ^f^	32.17 ± 0.34 ^d^	28.59 ± 0.40 ^f^

^1^ Y, ‘Jantarnyi’ with yellow fruits; C, ‘Koralovyi’ with coral fruits; R, ‘Podolski’ with red fruits; ^2^ I, single-step process of fermentation; II, two-step process of fermentation; ^3^ Values are expressed as the mean (*n* = 3) ± standard deviation. Mean values with different letters (a, b, c, etc.) within the same column are statistically different (*p* < 0.05).

**Table 3:** Iridoids and phenolic compounds content (mg/100 mL) in different vinegars from *Cornus mas* L.

Compound	RI	RII	CI	CII	YI	YII
Iridoids						
LA	140.71 ± 0.27 ^e,1^	178.46 ± 0.73 ^c^	175.54 ± 1.01 ^c^	190.75 ± 1.19 ^a^	158.38 ± 0.52 ^d^	185.07 ± 4.18 ^b^
S+Lo	15.24 ± 0.42 ^e^	34.72 ± 0.80 ^a^	19.83 ± 0.23 ^d^	27.38 ± 0.97 ^c^	15.63 ± 0.12 ^f^	29.41 ± 0.03 ^b^
Co.	17.20 ± 0.02 ^e^	30.30 ± 0.19 ^c^	22.49 ± 0.04 ^d^	35.04 ± 0.39 ^a^	10.42 ± 0.08 ^f^	32.92 ± 0.10 ^b^
Phenolic acids						
EA	0.87 ± 0.02 ^e^	1.99 ± 0.02 ^a^	1.13 ± 0.02 ^d^	1.69 ± 0.01 ^b^	0.62 ± 0.00 ^f^	1.52 ± 0.01 ^c^
Total CQA d	10.47 ± 0.00 ^e^	19.66 ± 0.24 ^b^	15.16 ± 0.15 ^d^	24.18 ± 0.27 ^a^	9.64 ± 0.05 ^f^	17.10 ± 0.07 ^b^
Total *p*-CA d	3.50 ± 0.00 ^f^	6.70 ± 0.43 ^d^	7.39 ± 0.01 ^c^	10.85 ± 0.02 ^a^	4.89 ± 0.06 ^e^	9.21 ± 0.01 ^b^
Anthocyanins						
Cy 3-gal	0.29 ± 0.02 ^b^	0.75 ± 0.03 ^a^	nd	nd	nd	nd
Cy 3-rob	0.19 ± 0.02 ^b^	0.41 ± 0.00 ^a^	nd	nd	nd	nd
Pg 3-gal	0.65 ± 0.04 ^b^	1.69 ± 0.02 ^a^	nd	nd	nd	nd
Pg 3-rob	0.20 ± 0.01 ^b^	0.30 ± 0.20 ^a^	nd	nd	nd	nd
Flavonols						
A 7-glu	1.80 ± 0.02 ^b^	3.35 ± 0.06 ^a^	nd	nd	nd	nd
Q 3-gal	0.32 ± 0.02 ^c^	0.58 ± 0.01 ^a^	0.39 ± 0.05 ^b^	0.59 ± 0.01 ^a^	0.19 ± 0.00 ^d^	0.34 ± 0.00 ^c^
Q 3-gluc	2.63 ± 0.02 ^e^	4.60 ± 0.05 ^b^	4.07 ± 0.05 ^c^	6.00 ± 0.05 ^a^	2.80 ± 0.01 ^d^	4.58 ± 0.00 ^b^
Kp 3-gal	1.38 ± 0.00 ^b^	2.53 ± 0.06 ^a^	0.10 ± 0.00 ^c^	0.17 ± 0.01 ^c^	nd	nd

LA, Loganic acid; S, Sweroside; Lo, Loganin; Co., Cornuside; EA, Ellagic acid; Total CQA d, total amount of caffeoylquinic acid derivatives; Total *p*-CA d, total amount of *p*-coumaric acid derivatives; Cy 3-gal, Cyjanidin 3-*O*-galactoside; Cy 3-rob, Cyjanidin 3-*O*-robinobioside; Pg 3-gal, Pelargonidine 3-*O*-galactoside; Pg 3-rob, Pelargonidine 3-*O*-robinobioside; A 7-glu, Aromadendrin 7-*O*-glucoside; Q 3-gal, Quercetin 3-*O*-galactoside; Q 3-gluc, Quercetin 3-*O*-glucuronide; Kp 3-gal, Kaempferol 3-*O*-galactoside. ^1^ Values are expressed as the mean (*n* = 3) ± standard deviation. Mean values with different letters (a, b, c, etc.) within the same row are statistically different (*p*-value < 0.05); nd, not detected.

**Table 4:** Description and symbols of vinegar production methods and Cornelian cherry fruits used in the experiment.

Symbol	Description	
I	Single-stage (spontaneous) alcoholic-acetic fermentation under controlled aerobic conditions.	Vinegar production method
II	Two-stage fermentation including first alcoholic fermentation with the use of *Saccharomyces bayanus—*Safspirit fruit yeast, followed by spontaneous acetic fermentation.	
Y	Yellow (‘Yantarnyi’ variety with yellow fruits)	Cornelian cherry variety/fruit color
C	Coral (‘Koralovyi’ variety with coral fruits)	
R	Red (‘Podolski’ variety with red fruits)	
